# Exploring adolescents’ perspectives on social media and mental health and well-being – A qualitative literature review

**DOI:** 10.1177/13591045221092884

**Published:** 2022-06-07

**Authors:** Anjali Popat, Carolyn Tarrant

**Affiliations:** 1 School of Medicine, University of Leicester, Leicester, UK; 2 Department of Health Sciences, University of Leicester, Leicester, UK

**Keywords:** Social media, mental health, wellbeing, adolescent perspective, social media addiction, connection, cyberbullying

## Abstract

Many quantitative studies have supported the association between social media use and poorer mental health, with less known about adolescents’ perspectives on social media’s impact on their mental health and wellbeing. This narrative literature review aimed to explore their perspectives, focusing on adolescents aged between 13 and 17. It reviewed qualitative studies published between January 2014 and December 2020, retrieved from four databases: APA Psychinfo, Web of Science, PubMed and Google Scholar. The literature search obtained 24 research papers. Five main themes were identified: 1) Self-expression and validation, 2) Appearance comparison and body ideals, 3) Pressure to stay connected, 4) Social engagement and peer support and 5) Exposure to bullying and harmful content. This review has highlighted how social media use can contribute to poor mental health – through validation-seeking practices, fear of judgement, body comparison, addiction and cyberbullying. It also demonstrates social media’s positive impact on adolescent wellbeing - through connection, support and discussion forums for those with similar diagnoses. Future research should consider adolescent views on improvements to social media, studying younger participants, and the impact of COVID-19 on social media use and its associated mental health implications.

## Introduction

‘Social media’ describes online platforms that enable interactions through the sharing of pictures, comments and reactions to content (Carr & Hayes, 2015). As most teenagers regularly use social media (Anderson & Jiang, 2018), studying its effects on their mental health and psychological wellbeing is vital. The term ‘psychological wellbeing’ reflects the extent to which an individual can live meaningfully according to their deeply held values (Waterman et al., 2010). Within this, the term ‘mental health’ was defined by WHO (2018) as a state of wellbeing in which an individual can flourish, through realisation of one’s potential, positive social interaction and contribution to society. Research in this field has been largely quantitative: providing evidence for the association between social media and mental health, but limited insight into the experiences and perceptions of adolescents of social media and its impact. This narrative review aims to synthesise recent qualitative research on adolescents’ perspectives of the effect of social media on their mental health and psychological wellbeing.

Mental health difficulties are becoming increasingly prevalent amongst young adults, accounting for 16% of disease in 10–19 year olds (WHO, 2020a, 2000b). Social and emotional wellbeing are key to an individual’s relationships and sense of belonging, whilst overall psychological wellbeing influences self-acceptance, personal growth and coping strategies (CDC, 2018). Numerous quantitative studies have supported the association between social media use and psychological issues (Keles et al., 2019), specifically increased rates of depression (Lin et al., 2016), anxiety (Dhir et al., 2018) and reduced self-esteem (Woods & Scott, 2016). In addition, exposure to self-harm content on social media has been linked to psychological harm and self-harm and suicidal ideation, particularly amongst vulnerable users (Arendt et al., 2019). This is alarming considering its integral role in teenage lives. ‘Psychological harm’ includes feeling threatened, intimidated and excluded by others; its effects depend on context and individual protective and vulnerability factors (Yoo & Smetana, 2019).

Reduced social media use has also been correlated with improved psychological outcomes (Hunt et al., 2018). Best et al.’s. (2014) systematic review evaluated quantitative and qualitative data on the effects of social media on adolescent wellbeing. Its benefits included social support, self-expression and access to online mental health resources, but significant negative aspects included social isolation and cyberbullying. Importantly, the purpose and context of social media use is crucial and supersedes the variable of ‘screen time’ which has been recognised to lack causal influence on psychological wellbeing (Kaye et al., 2020).

This review provides insight into the mechanisms through which social media can impact on mental health and psychological wellbeing, from the perspectives of adolescents themselves.

## Method

A systematic literature search and narrative synthesis were conducted. The review included articles from 2014 onwards. The SPIDER tool (Cooke et al., 2012) was used to design the literature search; inclusion and exclusion criteria are given in Table 1.

**Table 1.** Inclusion and exclusion criteria.

Inclusion criteria	Exclusion criteria
Adolescent perspective (13–17 years)	Children under 13 or older age groups (e.g. University students)
Qualitative studies Peer reviewed journal	Quantitative studies Correlational analysis Not available in English
Focus on social media	Internet use more broadly Social media use and the COVID-19 pandemic

**Figure 1.**
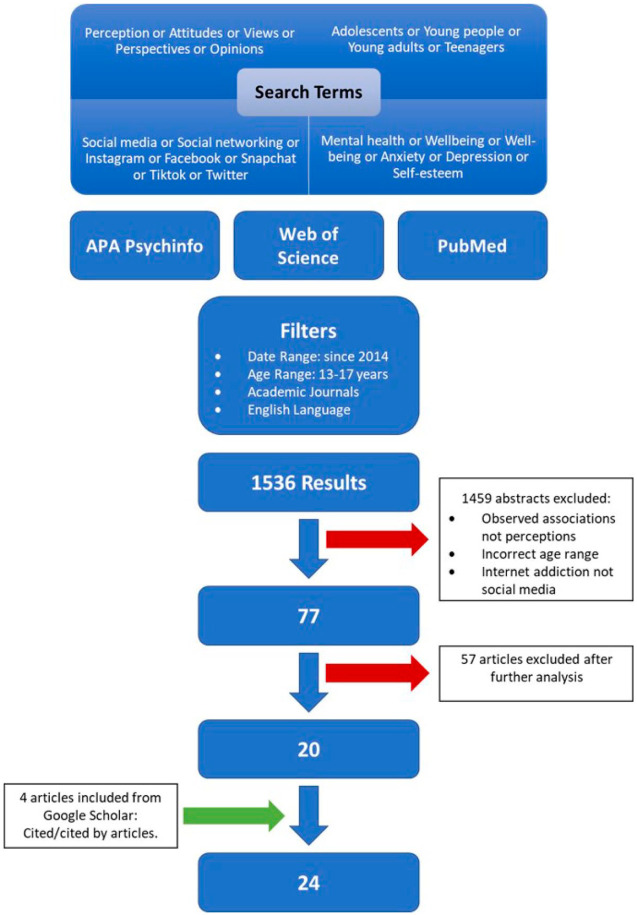
Flowchart of data collection.

Searches were conducted using APA PsychInfo, Web of Science Core Collection and PubMed. Search terms are shown in Figure 1. Initial search results were screened to exclude quantitative studies, and those about internet use more broadly. Studies of social media use during the COVID-19 pandemic were excluded due to the complexity of factors involved, including confounding factors for mental health. Papers that included a significant proportion of the 13–17 target age group but also included comparison to slightly younger or older age groups, were included, with analysis focused on the target age range within the study sample. Full text of 77 papers was reviewed and 57 excluded due to not meeting inclusion criteria. Twenty papers were selected for inclusion; an additional four papers were identified from searches of reference lists and citation searches (see Figure 1).

Findings were extracted and synthesised using thematic synthesis (Thomas & Harden, 2008). Qualitative findings from each article were open-coded and recurrent themes identified from comparison across codes. Mind-maps were used to develop and refine themes. Excel was used to organise key findings by theme, and to enable key wellbeing concepts in each theme to be extracted from the key findings in each theme. Through discussion between AP and CT, subsequent theme narratives were written, with regular reference back to the papers to adjust the themes accordingly and ensure inclusion of salient points. See Appendix A1 for a summary of articles contributing to each theme.

## Results

Five themes were identified, describing the mechanisms through which social media impacts on mental health and wellbeing: self-expression and validation; appearance comparison and body ideals; pressure to stay connected; social engagement and peer support; and exposure to bullying and harmful content. Themes and corresponding mental health/wellbeing concepts identified from included papers, with supporting quotes, are given in Table 2. Notably, adolescents reported a mixture of direct personal experiences and emotions associated with their own social media use, as well as general third-person views on its effects on the adolescent population. This is highlighted by the range of quotes, with some reflecting stances towards social media, and others describing effects of the platforms on their own mental health.

**Table 2.** Themes and corresponding wellbeing concepts, with supporting quotes from the papers.

Theme	Positive/Negative?	Corresponding wellbeing concepts	Supporting quotes
1) Self-expression and validation	Both	Self-esteem Social support Identity experimentation Social comparison Anxiety and fear of judgement Risky behaviour Self-acceptance Personal development	“When you’re changing your profile picture it’s like, “What are people going to think? What’s going to happen?” Are people going to comment or are they not going to comment?” (Calancie et al., 2017, p.5)“Everyone’s gotta be better than everyone and if you’re not better then you’re nothing” (Singleton et al., 2016, p. 398) “You can look back at all your old photos. . . see how you’ve developed” (Weinstein, 2018, p. 3611)
2) Appearance comparison and body ideals	Negative	Self-esteem Body image Social comparison Eating disorder relapse	“You want to be popular, you want people to like you, you want boys to like you” (Berne et al., 2014, p. 530) “If someone is insecure about their appearance, then people tend to use that. . .it’s a good weapon” (Berne et al., 2014, p. 529) “They have makeup artists, Photoshop, they have work done to them” (Burnette et al., 2017, p. 121)
3) Pressure to stay connected	Negative	Social pressure Social exclusion Emotional detachment Disconnection anxiety Sleep disruption Physical health issues	“Social media is like an online drug”(O’Reilly et al., 2018, p. 608) “Just think about all the things that I wouldn’t see . . .I feel like once you get it, you’re basically stuck” (Calancie et al., 2017, p. 8) “You’re always wondering ‘What’s everyone else doing?.. Should I be up?’ And then yeah- it affects my sleep” (Scott et al., 2019, p. 542)
4) Social engagement and peer support	Positive	Social support Social participation Connection Mental health recovery	“If I wasn’t on social networking sites, I wouldn’t know half the people I know. I definitely feel more confident talking to some people online. . .” (Kennedy & Lynch, 2016, p. 160) “You notice that there’s thousands all across the world in the same boat as you” (Singleton et al., 2016, p. 400) “‘Hundred-happy-day’ challenge. . .every day you took a photo about what made you happy” (O’Reilly et al., 2018, p. 984)
5) Exposure to bullying and harmful content	Negative	Emotional distress Anxiety Suicidal thoughts Self-esteem Lack of control Injustice Social exclusion and threats to belonging Loneliness and isolation	“It’s a dangerous place social media” (O’Reilly, 2020, p. 203) “People take advantage. . .being anonymous. . .target you with loads of rude comments and that can make you feel really insecure” (O’Reilly et al., 2018, p. 607) “If you’re exposed to a lot of stuff about self-harm and mental illness, it might affect you mentally as well” (O’Reilly, 2020, p. 203)

### Theme 1: Self-expression and validation

Across studies, adolescents reported social media’s potential to encourage self-expression. They constructed their profiles to reflect their ‘best self’ (Kennedy & Lynch, 2016). Although potentially liberating, many admitted the importance of others’ opinions. Peer feedback led to cycles of modifying self-presentation (Kennedy & Lynch, 2016; Singleton et al., 2016), generating excitement when they received ‘Likes’^1^ from other users (Calancie et al., 2017). However, this was short-lived; they compulsively checked how many ‘Likes’ and ‘Comments’^2^ they received (Calancie et al., 2017; Radovic et al., 2017). This need for validation was accepted as a negative but integral part of social media use (Singleton et al., 2016). Adolescents frequently compared their numbers of ‘Likes’ with peers; receiving fewer could impact negatively on their self-esteem. Those with pre-existing anxiety were most heavily affected (Singleton et al., 2016; Calancie et al., 2017).

‘Selfie’^3^ posting was common. Girls experienced higher expectations to post but had stronger privacy concerns; boys felt selfie posting could enhance popularity, but highlighted that their desire to attract ‘Likes’ could sometimes fuel posting with less consideration of online privacy and appropriateness of the content (Boursier et al., 2020). Although selfie-posting was frequently used to seek validation, there were tacit rules about selfie-posting that created hypervigilance; users should post enough to feel seen, but not so much to risk judgement (Kennedy & Lynch, 2016; Singleton et al., 2016).

Burnette et al. (2017) found evidence, in their focus group study of 12–14 year old girls, that the negative impact of approval-seeking practices and judgement on self-esteem could be buffered. An extra-curricular school club nurtured self-acceptance through teaching body positivity and diversity, and helped adolescents learn to use social media in healthier ways, for example, sharing photos from social events rather than individually-focused selfies. Additionally, Weinstein (2018) in a study of 14–18 year olds, identified that some adolescents had found ways of using social media for self-expression whilst avoiding the potential negative dynamics. Creating ‘Spam accounts’,^4^ where only close friends could view posts, enabled comfortable expression without anxiety of others’ judgement. They also described creating private accounts for themselves: posting pictures as reflective journals for self-validation and a sense of personal development.

### Theme 2: Appearance comparison and body ideals

On photo-sharing platforms, where visual content predominated, appearance was highly valued. ‘Perfect’ images receiving hundreds of ‘Likes’ symbolised popularity. Adolescent girls felt that ‘Likes’ were confirmations of meeting specific body ideals, rooted in media representation of beauty (Berne et al., 2014). Picture-based social media was viewed as particularly damaging to self-esteem through appearance comparison.

Additionally, photo-shopped images fuelled pre-existing body expectations. Teenagers knew celebrities edited their pictures but still made comparisons between their own bodies and images of the unattainable ‘perfect’ body. This reduced their self-esteem significantly, inducing feelings of unworthiness, and negatively impacting their body image (Burnette et al., 2017; O’Reilly et al., 2018). The prevalence of such images online made them difficult to evade. Images of ‘perfect bodies’ were deemed particularly harmful to those with past eating disorders (Radovic et al., 2017); viewing this content could lead to relapsing into old thinking patterns. Teenagers also mentioned the potential of ‘perfect body’ posts to trigger the development of disorders like anorexia, through people striving to lose weight to meet body ideals (Radovic et al., 2017).

As well as the damaging impact of body images encountered on social media, cyberbullying targeting body shape added to appearance anxiety. This frequently involved weight-shaming, admitted by some males as an effective way of lowering girls’ self-esteem (Berne et al., 2014). Although girls felt more frequently scrutinised, boys also experienced criticism. For example, some boys described how posting ‘selfies’ in unique clothing, or posting meaningful captions, had triggered comments questioning their sexuality which they experienced as negative (Berne et al., 2014). Nevertheless, many highlighted that damaging effects could be buffered, for example, through body positivity education (Burnette et al., 2017). This could reduce compulsion to compare, through awareness of the artificial nature of online posts, and developing self-confidence.

### Theme 3: Pressure to stay connected

Adolescents frequently described how online interaction dominated and was integral to everyday life. They felt pressure to stay connected, for example, participating in ‘streaks’, whereby users sent each other social media content every day to sustain the number of days of contact. Breaking these norms was considered rude, so users continued to engage despite the burden (O’Reilly et al., 2018). The thought of disconnecting induced fear: being unaware of what was occurring online risked offline social exclusion (Kennedy & Lynch, 2016; Scott et al., 2019; Thomas et al., 2016). This fuelled compulsive use; adolescents described the reflex action of picking up their phones and scrolling through several social media apps in turn (O’Reilly et al., 2018; Scott et al., 2019).

The pressure to stay connected to social media could reduce offline social engagement. Many felt social media diminished their quality time with family and friends, resulting in emotional detachment (Mulisa & Getahun, 2018). Adolescents experienced problems with household family dynamics when phone use was valued over face-to-face interaction (Kennedy & Lynch, 2016). Additionally, some reported physical health problems of screen use, like headaches, blurred vision and sleeping difficulty (Smahel et al., 2015). Flicking through apps at bedtime caused heightened cognitive arousal, and this combined with the artificial light made falling asleep difficult.

Thomas et al. (2016) explored the hypothetical situation of unplugging from social media for 24 hours; participants felt anxious and uneasy about not informing others that they had disconnected. Those with previous experience of unplugging found it much easier, and produced greater activity ideas to engage with whilst disconnected. Adolescents mostly talked in the third person about problems disconnecting from social media, suggesting that teenagers were normalising their own behaviour in contrast with others who might be ‘addicted’ to social media. This could potentially enable them to avoid confronting their own levels of social media use.

### Theme 4: Social engagement and peer support

Adolescents described how social media could positively contribute to wellbeing, through supporting social engagement and enabling access to peer support. By tweeting, texting and posting, friendships blossomed and strengthened. Individuals highlighted the importance of an online presence in enabling social interaction (Best et al., 2015; O’Reilly, 2020; Radovic et al., 2017; Thomas et al., 2016) They described the ease of forming connections online, with less pressure than in-person meetings (Kennedy & Lynch, 2016). Online friendships fostered increased support and positively correlated with wellbeing (Best et al., 2015). Adolescents valued the number of ‘friends’^5^ or ‘followers’^6^ they had: higher numbers boosted self-esteem, acting as validation of their demonstrated popularity. But although bigger networks felt comforting, teenagers did not always feel that their online friends cared or would offer them support. Most felt that offline or ‘real life’ friends held greater value (Best et al., 2015).

As well as providing a platform for social engagement, social media initiatives could promote positive mental health. For instance, teenagers in O’Reilly et al. (2018) described participating in ‘challenges’ to improve their wellbeing, like posting a photo everyday of something personally uplifting. They then ‘tagged’^7^ friends to participate, spreading positivity. Social media could also be a place for discussion around mental health. Viewing celebrities’ recovery stories reduced isolation amongst individuals facing similar experiences, (O’Reilly et al., 2018) and enabled formation of support networks for those with related diagnoses. Educational resources on maintaining positive mental health were accessible and spread quickly online.

Several studies explored the value of online discussion forums, moderated by healthcare professionals, for those suffering from physical and mental health problems (Fergie et al., 2016; Kendal et al., 2017). Users valued the massive support network provided, and having a safe space to share their feelings. Importantly, forums helped individuals overcome fears of seeking professional help, with reassurance from others that this had aided in their recovery (Kendal et al., 2017). Similarly, social media could be an effective platform for education around sensitive topics (Aragao et al., 2018).

Young people highlighted a need for better guidance at school on benefitting from the positive side of social media - finding trustworthy information and appropriate groups to join (O’Reilly et al., 2018).

### Theme 5: Exposure to bullying and harmful content

Despite social media’s benefits, adolescents frequently reported exposure to bullying and harmful content, which could have significant negative impacts on mental health and wellbeing. Anonymity enabled targeting – bullies and trolls^8^ could post hurtful comments without punishment because their identity remained hidden (Singleton et al., 2016; O’Reilly et al., 2018). ‘Cyberbullying’, including name-calling and public humiliation, caused teenagers anxiety (Calancie et al., 2017), but there was widespread acceptance that they must ignore or tolerate it (O’Reilly et al., 2018; Wang et al., 2019).

Teenagers who had experienced cyberbullying reported its significant impact on their mental health, describing feelings of distress, confusion and isolation (Smith et al., 2017; O’Reilly et al., 2018). Whilst aware of the injustice, they were unsure of where to turn (Wang et al., 2019). Repeated instances were incredibly damaging, sometimes causing suicidal thoughts (O’Reilly, 2020). Those targeting others admitted intentions to lower another’s self-esteem, fuelled by jealousy or discontent with their own life (Berne et al., 2014).

Adolescents also described subtle online exclusion, including the lack of invitation to events and unresponsiveness to messages. Smith et al. (2017) found that when adolescents were randomly allocated to ‘inclusive’ newsfeeds (many friends, message reciprocity and strong reactions to posts) they experienced feelings of connection, whereas those exposed to ‘exclusive’ newsfeeds (rapidly declining friends’ lists and lack of event invitations) felt high levels of threats to their sense of belonging.

Privacy concerns were also common; most platforms had ‘private’ setting options, but adolescents reported that others could share pictures of them without consent. Lack of control induced anxiety, worsened by the permanency of uploaded content (Wang et al., 2019). Furthermore, some reported instances of account hacking and impersonation, inducing fear as others could post inappropriate content under their identity (Weinstein & Selman, 2014).

As well as online bullying, adolescents frequently described exposure to damaging content, including self-harm posts, which were particularly problematic for those with a history of mental illness (Singleton et al., 2016; Radovic et al., 2017; O’Reilly, 2020). Participants also described how viewing scary or violent images online, often unintentionally, disturbed their sleep: cognitive salience caused graphic images to replay in their minds (Smahel et al., 2015). Social media also prevented escape from distressing news; they felt trapped in the rapidly updating online world (Weinstein, 2018). Additionally, teenagers admitted to posting impulsively when upset or angry, termed ‘stress posting’, often causing conflict that frequently extended offline (Radovic et al., 2017).

## Discussion

This review demonstrates the complex effect of social media on adolescent wellbeing, from the perspectives of teenagers themselves. On the one hand, social media fosters connection and support: online connections are a form of social capital, providing support and validation. Social media enables teenagers to learn from others about dealing with difficult situations and mental health problems. In addition, moderated discussion forums encourage open conversations around difficult topics, reducing isolation and aiding recovery of mental health difficulties.

On the other hand, social media use can impact negatively on wellbeing and mental health, damaging self-esteem through experience of judgement, attention to markers of popularity, and appearance comparison. Posting without considering privacy or appropriateness, and ‘stress’ posting could have negative longer term consequences. Teenagers highlighted concerns about social media impacting negatively on their real life relationships, and causing anxiety and sleep disruption. Teenagers also reported the well-documented negative consequences of social media use of cyberbullying, online exclusion, and the impact of viewing distressing content. Despite the strong association between cyberbullying and face-to-face bullying, research has argued that the anonymous nature of cyberbullying enables more extreme levels of victimisation, and its repetitive nature has a more intense impact on the individual (Accordino & Accordino, 2011).

Importantly, there was significant interplay between the five emerging themes, reflecting the complexity of factors at play. For instance, Singleton et al. (2016) found that although online connection was positive, it resulted in the need to stay informed about others’ lives, which led to compulsive use of the sites in fear of not knowing. Similarly, in the study by O’Reilly et al. (2018), adolescents admitted that the positive connection aspect quickly turned into reliance on social media to stay connected, fuelling addiction. This issue of disconnection anxiety was also highlighted in other studies (Scott et al., 2019; Thomas et al., 2016). Furthermore, although self-expression was reported as hugely positive, there was constant fear of judgement and strict adherence to ‘virtual norms’ to protect against this (Kennedy & Lynch, 2016; Weinstein, 2018). In addition, the expectation to share was a heavy burden; some would prefer to keep their lives private and only accept close friends on their profiles, but feared judgement from others regarding numbers of online friends (Calancie et al., 2017). In this way, the reported positive and negative aspects of social media had an inter-related effect on the way in which adolescents used and felt about the sites.

Setting these findings in the context of adolescent cognitive and social development helps provide insight into the mechanisms that shape the impact of social media on mental health and wellbeing during the teenage years. Adolescence is a period of significant developmental brain changes. The pre-frontal cortex, involved in rational decision-making, has not fully formed. Therefore, teenagers often rely on their amygdala, an emotional processing zone, when making choices. Furthermore, the nucleus accumbens, regulating dopamine-modulated reward, is hyperactive (Casey et al., 2008). This makes adolescents particularly vulnerable to impulsive decision-making, posing risk on social media platforms where content is shared with a single click. Additionally, during this period, neural connections are fine-tuned through synaptic pruning, enhancing the effect of social media content in reinforcing certain thought patterns (Selemon, 2013) – which can include negative thoughts about self-image and self-worth. Adolescents also reported that heavy social media use disrupted their sleep, potentially impairing cognitive and emotional functioning (Walker, 2018). However, sleep quality has also been correlated with mental health and other factors (Milojevich & Lukowski, 2016), hence there is inter-relation between these variables, and causality cannot be inferred.

Perhaps most importantly, in term of understanding the impact of social media on mental health, is the nature of social development that happens during adolescence. Erikson’s Theory of Psychosocial Development (1950) suggests that adolescence involves the struggle between fitting in and standing out, causing identity experimentation; social media provides a platform on which this struggle is played out. Lewin’s Field Theory (1951) points to adolescence as being an important transition where the dynamics between social environment and psychological change dictate behaviour. Social media’s virtual social circles act as digital social environments that can powerfully shape thoughts and behaviours.

This raises issues surrounding digital rights, explored extensively by Livingstone and Third (2017). Due to the immature stage of psychological development during childhood and adolescence there remains concern over their identical digital rights to adults (Lister, 2008). It has been argued that online activity requires a high level of responsibility, such as protecting the self and others online, a heavy demand before adulthood (Third & Collin, 2016). Furthermore, digital traces of online acts leave adolescents exposed to judgement, potentially interfering with the self-expression that is key to enhancing development (Bulger et al., 2017). Children’s rights may be the responsibility of their primary caregiver, but once access to online platforms has been granted, protection should continue due to the array of harms that could be encountered online (Livingstone & Third, 2017). Crucially, this protection needs to be balanced with enabling participation on digital platforms, which has been supported in aiding social development (Swist et al., 2015). In this way, children and adolescents should feel confident to reap the benefits of the digital world whilst feeling adequately protected.

A significant challenge is the view of adolescents as a homogeneous population. This review studied adolescents aged between 13 and 17, and the huge diversity within this is recognised, with digital impact being multi-factorial across many psychosocial elements. Individuals will vary in their digital literacy skills, which are important for safe use of online resources including social media (Sonck et al., 2011). Livingstone (2013) has emphasised that online risk does not imply subsequent harm, or equal harm to all users. The effect is dependent on the complex interplay between individual protective and vulnerability factors, and specific environmental exposures, resulting in differing impact between individuals. For instance, they identified that high self-esteem and effective parental management of internet use were protective factors, whilst pre-existing anxiety or depression made individuals more vulnerable to online harm. Furthermore, individuals with higher digital resilience could better recognise and manage online risk and hence buffer against potential harm. Importantly, those more vulnerable to offline risk were more likely to be vulnerable to online risk (Bradbook et al., 2008). In today’s age, these variables are blended as offline and online behaviour are strongly interlinked and hence their effects have huge cross-over. More research is therefore needed to enable better understanding of the relationship between offline life circumstances and online experiences.

This review focused on qualitative research, which provides rich insight into the perspectives of adolescents themselves. Focus groups and interviews enable open discussion of personal experiences and sensitive topics. In terms of limitations, most studies did not include diverse populations, being weighted towards one gender (Singleton et al., 2016) or geographical location (O’Reilly, 2020). In addition, adolescents had varied understandings of ‘mental health’, with some attaching negative connotations to the term (O’Reilly et al., 2018), meaning that positive impacts of social media may be under-reported.

There were differences in methodology between papers, with some using solely one method and others using mixed methods. Ten studies used focus groups; seven used semi-structured interviews; eight used online qualitative questionnaires either alone or in combination with other qualitative methods, and the remaining two studies analysed online data, such as posts of opinions and experiences of social media. There was also variability in the samples used between studies, in terms of numbers of participants, ratio between genders, and other factors such as level or nature of schooling. Those with larger study samples and focus groups of target age range provided more valuable insight into this review, as participants could speak more freely and share ideas to fuel discussion within groups. Additionally, non-verbal cues could be analysed. Conversely, although studies analysing online posts of adolescent opinions provided extra anonymity, self-reporting of age online interferes with the reliability of the findings. There is also the issue of bias as those posting their opinions on networking sites have already chosen to engage with social media and could be more likely to highlight its positive aspects.

The heterogeneity between studies, particularly in terms of study design, enabled a wide variety of data to be collected and analysed, thus minimising the level of internal bias within the data sets. However, it is acknowledged that the variability between studies could result in uneven influence on the conclusions made.

Research to date has focused on adolescent and adult use of social media; future studies should analyse the impact of social media on younger children, as many younger children already use social media and are forming usage patterns as well as being exposed to negative impacts. Furthermore, research should focus on interventions to reduce the negative impacts, including suggestions from adolescent users themselves. These include early school education surrounding responsible social media use, and teaching body positivity and self-acceptance to children and teenagers of all ages (Burnette et al., 2017). Removal of the ‘Like’ button could reduce approval-seeking practices; many adolescents felt that compulsive monitoring of feedback on their posts was fuelled by the desire to fit in, and triggered anxiety (Calancie et al., 2017; Kennedy & Lynch, 2016; Singleton et al., 2016; Weinstein, 2018).

Lastly, future studies should be conducted after the COVID-19 pandemic, researching differing usage during national lockdowns and its associated impact on wellbeing. Exaggeration of both the positive connection aspects, and negative addiction aspects, would be likely. In this way, the effects of social media during an already challenging period could be assessed.

## Conclusion

In conclusion, this review highlighted the complex impact of social media on adolescent wellbeing. Their perspectives enabled in-depth understanding of the reasoning behind the positive and negative effects of social media usage on mental health. Looking forward, educational interventions and social media alterations could help buffer against the negative effects.

## Appendix

**Appendix A1.** Themes and corresponding papers.

Self-expression, judgement, validation	Appearance anxiety and body ideals	Addiction	Connection and support	Dangers of social media
Kennedy & Lynch (2016)	Berne et al. (2014)	Smahel et al. (2015)	Weinstein & Selman (2014)	Berne et al. (2014)
Singleton et al. (2016)	Burnette et al. (2017)	Kennedy & Lynch (2016)	Best et al. (2015)	Weinstein & Selman (2014)
Calancie et al. (2017)	Radovic et al. (2017)	Singleton et al. (2016)	Kendal et al. (2017)	Smahel et al. (2015)
Burnette et al. (2017)	O’Reilly, Dogra, Whiteman, et al. (2018)	Thomas et al. (2016)	Fergie et al. (2016)	Singleton et al. (2016)
Radovic et al. (2017)	Boursier et al. (2020)	Calancie et al. (2017)	Kennedy & Lynch (2016)	Calancie et al. (2017)
Weinstein (2018)	O’Reilly (2020)	Mulisa & Getahun (2018)	Singleton et al. (2016)	Radovic et al. (2017)
		O’Reilly, Dogra, Whiteman, et al. (2018)	Thomas et al. (2016)	Smith et al. (2017)
		Scott et al. (2019)	Calancie et al. (2017)	Mulisa & Getahun (2018)
		O’Reilly (2020)	Naslund et al. (2017)	O’Reilly et al. (2018)
			Radovic et al. (2017)	Weinstein (2018)
			Aragao et al. (2018)	Wang et al. (2019)
			Mulisa & Getahun (2018)	O’Reilly (2020)
			O’Reilly, Dogra, Hughes et al., 2018	
			Weinstein (2018)	
			Nikolaou et al. (2019)	
			O’Reilly (2020)	

## Notes

1. A quick way to show approval through the click of a button (Stec, 2015).

2. A message response to content on social networking sites (Stec, 2015).

3. A self-portrait usually taken using the reverse camera screen on a smartphone (Stec, 2015).

4. Secondary private accounts created to share personal content with exclusive groups of people (Placide, 2020).

5. Online connections who can view and interact with one’s content (Stec, 2015).

6. Those who subscribe to an account to obtain regular updates regarding the account (Stec, 2015).

7. Linking another user’s profile who may be relevant to the content (Stec, 2015).

8. Individuals who post controversial comments online to intentionally provoke emotional reactions in others (Stec, 2015).
